# Blood Donation and Colorectal Cancer Incidence and Mortality in Men

**DOI:** 10.1371/journal.pone.0039319

**Published:** 2012-06-22

**Authors:** Xuehong Zhang, Jing Ma, Kana Wu, Andrew T. Chan, Charles S. Fuchs, Edward L. Giovannucci

**Affiliations:** 1 Channing Laboratory, Department of Medicine, Harvard Medical School, Brigham and Women’s Hospital, Boston, Massachusetts, United States of America; 2 Department of Nutrition, Harvard School of Public Health, Boston, Massachusetts, United States of America; 3 Gastrointestinal Unit, Harvard Medical School, Massachusetts General Hospital, Boston, Massachusetts, United States of America; 4 Department of Medical Oncology, Dana-Farber Cancer Institute, Harvard Medical School, Boston, Massachusetts, United States of America; 5 Department of Epidemiology, Harvard School of Public Health, Boston, Massachusetts, United States of America; Baylor College of Medicine, United States of America

## Abstract

**Background:**

Although blood donations may reduce body iron stores, to date, prospective data on frequent blood donation and colorectal cancer risk are limited.

**Methodology/Principal Findings:**

We tested whether frequent blood donation is associated with a lower risk of colorectal cancer in the Health Professionals Follow-up Study. We prospectively followed 35,121****men who provide the information on lifetime number of blood donations in 1992 through 2008. Serum ferritin levels were measured in a random sample of 305 men. Cox proportional hazard regression models were used to calculate the multivariable relative risks (RRs, 95%CIs) after adjusting for age and other established colorectal cancer risk factors. We documented 684 incident colorectal cancer cases and 224 deaths from colorectal cancer. The mean serum ferritin levels varied from 178 µg/L for men who did not donate blood to 98 µg/L for men who had at least 30 donations. Age-adjusted results for both incidence and mortality were essentially the same as the multivariable-adjusted results. Comparing with non-donors, the multivariable RRs (95%CIs) for colorectal cancer incidence were 0.92 (0.77, 1.11) for 1–5 donation, 0.85 (0.64, 1.11) for 6–9 donations, 0.96 (0.73, 1.26) for 10–19 donations, 0.91 (0.63, 1.32) for 20–29 donations, and 0.97 (0.68, 1.38) for at least 30 donations (*P*_trend_ = 0.92). The multivariable RRs for colorectal cancer mortality were 0.99 (0.72, 1.36) for 1–5 donation, 0.93 (0.57, 1.51) for 6–9 donations, 0.85 (0.50, 1.42) for 10–19 donations, and 1.14 (0.72, 1.83) for at least 20 donations (*P*_trend_ = 0.82). The results did not vary by cancer sub-sites, intake levels of total iron, heme iron, or family history of colorectal cancer.

**Conclusions/Significance:**

Frequent blood donations were not associated with colorectal cancer incidence and mortality in men. Our results do not support an important role of body iron stores in colorectal carcinogenesis.

## Introduction

Iron is an essential mineral but excessive iron has been hypothesized to influence colorectal carcinogenesis possibly through forming reactive oxygen species, as shown *in vitro* studies. [1], [2] In addition, given its abundance in food sources and widespread use of iron supplementation, the iron and colorectal cancer hypothesis warrants further investigation. To date, epidemiological studies of colorectal cancer risk in relation to iron intakes or markers of body iron stores have yielded conflicting results. [3], [4], [5], [6], [7], [8], [9], [10], [11], [12], [13] The inconsistent findings may partly be explained by the use of non-specific measures of body iron stores. [2], [9] This limitation could be addressed by using history of blood donation as a marker of body iron levels. [2] In men, body iron stores can be halved through the donation of 1 unit per year (U/yr). [14] As shown earlier, [2], [15] serum ferritin level is a reliable measure of body iron stores in healthy individuals compared to other measures such as serum transferrin and total iron binding capacity. The hypothesis that the depletion of body iron stores decreases colorectal cancer risk can be tested by comparing colorectal cancer risk between frequent blood donors and non-donors.

To the best of our knowledge, three studies [16], [17], [18] evaluated cancer risk among blood donors but the results may mainly reflect healthy donor effects and none estimated the dose-response relationship with the number of blood donations. One recent study in Swedish and Danish blood donors estimated iron loss resulting from blood donations and found little support for any important association between blood donation and overall cancer risk (no separate estimates for colorectal cancer due to the small numbers). [19] However, factors that do or may influence cancer risk including smoking, alcohol consumption, diet, body mass index, and physical activity were not available in that study. [19] The aim of this study is to examine the association between blood donation and colorectal cancer incidence and mortality in the Health Professionals Follow-up Study (HPFS). We hypothesized that frequent blood donation is associated with a lower risk of colorectal cancer.

## Materials and Methods

### Study Population

The HPFS is a prospective cohort study that was initiated in 1986 when 51,529 U.S. male professionals who aged 40 to 75 years were enrolled. [20] Participants have been mailed questionnaires every 2 years since 1986 to collect data on demographics, lifestyle factors, medical history, and disease outcomes. The follow-up rate has been greater than 90%. This study was approved by the Human Subjects Committee of the Harvard School of Public Health. As approved by the committee, return of the questionnaires was considered to imply informed consent and we also obtained written consent from each participant to obtain and review medical records. We began our analysis in 1992 when 46,213 participants returned the questionnaire in which blood donation history were queried. We excluded participants who died before 1992 (n = 11), or participants with a history of cancer (n = 3,681; except for non-melanoma skin cancer), or ulcerative colitis (n = 398) in 1992. We further excluded participants with missing data on blood donation (n = 7,002), which left 35,121 men for the analysis. Men who did not answer the blood donation question did not differ substantially from respondents according to age, body mass index, physical activity, endoscopy screening, family history of colorectal cancer, alcohol consumption, and major dietary factors (data not shown).

### Identification of Incident Colorectal Cancer Cases and Deaths

Participants reported cancer and other disease outcomes on the biennial questionnaires. Researchers were given permission by the study participants to obtain medical records and pathological reports. Researchers were blinded to exposure information and reviewed the medical records to abstract information on anatomic location, stage, and histological type of the cancer. Colon cancer and rectal cancer were defined according to the International Classification of Diseases, Ninth Revision (ICD-9). [21] Colon cancer was further classified into proximal colon cancers (neoplasms from the cecum to the splenic flexure) and distal colon cancers (neoplasms in the descending and sigmoid colon). Rectal cancer was defined as that occurring in the rectosigmoid or rectum. [21] A total of 684 incident colorectal cancer cases were documented in this study. Deaths were identified from state vital statistics records, the National Death Index, reported by the families, and the postal system. Cause of death was identified from death certificates or review of medical records. We documented 224 deaths from colorectal cancer in this analysis.

### Assessment of Blood Donation and Serum Ferritin

In 1992, the participants were asked to report their blood donation history (never, 1 to 5, 6 to 9, 10 to 19, 20 to 29, 30 to 59, 60 to 89, and ≥90 blood donations) during the past 30 years. We grouped participants with 30 to 59, 60 to 89, and ≥90 blood donations into 1 category (i.e., ≥30) because only relatively few men donated >30 U of blood. Serum ferritin levels were measured in a random sample of 123 men in 1986. [22] Additional samples from another182 men, who provided blood in 1994, were obtained in a control group of a nested case-control study of Parkinson disease (Dr. Xiang Gao, personal communication, 2010). Given similar serum ferritin levels in these two samples (*P*-value for test for difference = 0.07), we combined them to examine whether the serum ferritin levels differ by number of blood donations. CVs for the assays were <8%.

### Assessment of Dietary and Non-dietary Lifestyle Factors

Information on usual dietary intake over the past year was first assessed using a validated 131-item food frequency questionnaire (FFQ) in 1986 and every 4 years thereafter. [20] Nine possible frequency choices were available, ranging from “almost never” to “6 or more times per day”. Nutrient intakes were calculated by multiplying the frequency of each food consumed and the nutrient content of specified portion sizes. These dietary factors included total iron, dietary iron, heme iron, iron supplement use; and information on red meat, processed meat, alcohol, folate, calcium, vitamin D, fruits, and vegetables was also collected from the baseline and in subsequent FFQs. In addition, we inquired about potential colorectal cancer risk factors such as height, body weight, physical activity (MET-hrs/wk), cigarette smoking, family history of colorectal cancer, and aspirin use in the biennial questionnaires.

**Table 1:** Age-standardized risk factors for colorectal cancer in relation to lifetime number of blood donation in the Health Professionals Follow-up Study.

	No. of blood donations					
	0 (n = 11554)	1–5 (n = 11699)	6–9 (n = 4082)	10–19 (n = 3811)	20–29 (n = 1731)	≥30 (n = 1817)
Age, mean (SD), y	61.1(9.9)	59.3(9.5)	58.0(9.1)	57.9(8.8)	57.9(8.8)	58.8(8.4)
Body mass index(kg/m^2^), mean (SD)^1^	25.4(3.2)	25.6(3.1)	25.7(3.1)	25.9(3.1)	26.1(3.3)	26.0(3.3)
Physical activity (MET-hrs/wk), mean(SD)^2^	28.3(28.6)	28.7(27.4)	30.3(28.5)	29.7(26.8)	30.0(27.1)	30.8(28.3)
Family history of colorectal cancer in a parentor sibling (%)	12	13	13	12	12	13
Former or current smokers (%)	50	51	51	52	50	49
Regular aspirin use (%)^3^	43	44	46	46	46	45
Multivitamin use (%)	43	43	43	42	43	42
Ever had a colonoscopy or sigmoidoscopybefore 1992 (%)	44	47	46	48	46	47
Dietary intakes^4^, mean (SD)						
Total energy intake(kcal/d)	1923(598)	1920(590)	1923(591)	1927(594)	1974(594)	1967(590)
Total carbohydrates (g/d)	249(44)	248(43)	247(41)	245(42)	245(40)	245(39)
Total fat(g/d)	68.5(14.4)	68.7(13.9)	68.9(13.6)	69.7(13.8)	70.1(13.2)	70.3(12.9)
Total protein(g/d)	90.8(16.4)	90.7(15.7)	90.6(15.4)	91.2(15.5)	90.9(15.3)	91.0(15.2)
Fruits and vegetables (servings/d)	5.5(2.6)	5.6(2.6)	5.6(2.4)	5.6(2.6)	5.7(2.5)	5.6(2.5)
Dietary fiber (g/d)^5^	22.3(7.7)	22.4(7.3)	22.4(7.2)	22.1(6.9)	22.0(6.9)	22.2(7.2)
Alcohol(g/d)	10.1(14.8)	10.3(14.3)	10.5(14.4)	10.4(13.7)	10.3(13.7)	9.9(13.8)
Total calcium (mg/d)^5^	905(409)	904(388)	920(382)	926(390)	914(374)	932(371)
Total vitamin D (IU/d)^5^	438(301)	436(295)	432(293)	432(294)	419(276)	427(275)
Total folate (µg/d)^5^	507(267)	508(268)	500(259)	500(263)	497(249)	490(240)
Total iron (mg/d)^5^	19.6(12.6)	19.5(12.4)	19.2(11.7)	19.2(12.0)	19.3(12.6)	19.8(12.9)
Heme iron (mg/d)^5^	1.2(0.5)	1.2(0.5)	1.2(0.5)	1.2(0.5)	1.2(0.4)	1.2(0.4)
Iron supplement use (%)	2	3	2	3	3	3
Beef, pork, or lamb as a main dish(servings/wk)	1.8(1.5)	1.7(1.4)	1.8(1.4)	1.8(1.4)	1.9(1.4)	1.9(1.6)
Processed meat(servings/wk)	1.1(1.6)	1.1(1.6)	1.1(1.5)	1.2(1.6)	1.2(1.5)	1.3(1.6)
Serum ferritin levels (µg/L)^6^,mean (SD)	178 (130.6)	173(124)	162(120)	129(93)	102(56)	98(67)

1 Body mass index was calculated as weight in kilograms divided by the square of height in meters.

2 MET denotes metabolic equivalent. MET-hours  =  sum of the average time/week spent in each activity x MET value of each activity.

3 Regular aspirin user was defined as consumption of 2 or more 325-mg tablets per week. Non-regular user was defined otherwise.

4 Intakes were estimated with food-frequency questionnaire in 1990 except for iron supplement use from the 1992 questionnaire.

5 Nutrient values were energy-adjusted intake.

6 The serum ferritin levels were measured in a random sample of 305 men in HPFS.

### Statistical Analyses

We calculated person-time for each participant from the 1992 questionnaire return to the date of death, colorectal cancer diagnosis, or the end of follow-up (January 1, 2008), whichever came first. We used a Cox proportional hazards regression model [23] to calculate hazards ratios (relative risks or RRs) and 95% confidence intervals (CIs) and adjusted simultaneously for age (in months) and year of questionnaire return. In addition to age adjustment, in the second model, we adjusted for established non-dietary risk factors, which were queried in 1992 questionnaire. In the third model, we adjusted for dietary factors (see Table 2 footnote for these variables). We used the 1990 questionnaire for the dietary factors because no food frequency questionnaire was administered in 1992. We used median values of blood donation categories and entered these values as continuous variables to conduct trend tests. We observed no violation of the proportional hazard assumption based on the likelihood ratio test that compared the model with and without the interaction terms between blood donation and age or follow-up time. Given that some factors or conditions may potentially influence body iron stores, [24] we evaluated whether the association with the number of blood donations varied by age (<65, ≥65 y), alcohol consumption (non- to low drinker [<10 g/d], moderate- to heavy-drinker [≥10 g/d]), and total iron and heme iron intakes (<median, ≥median). In addition, we conducted sensitivity analyses restricted to men without gastrointestinal bleeding and inflammatory conditions such as myocardial infarction, stroke, coronary artery bypass graft angina or rheumatoid arthritis. Further, we assessed whether the association was modified by common colorectal cancer risk factors including endoscopy screening (no, yes), body mass index (<25, ≥25 kg/m^2^), physical activity (<30, ≥30 MET-hrs/wk), and family history of colorectal cancer (no, yes). The information on these factors was from the 1992 questionnaire.

**Table 2:** The relative risks of colorectal cancer and sub-sites according to lifetime number of blood donations in the Health Professionals Follow-up Study (1992–2008).

		Number of blood donations						P for trend
		0	1–5	6–9	10–19	20–29	≥30	
**Colorectal cancer**	No of Cases (n = 684)	257	220	68	69	33	37	
	Model 1^1^	1.0 (reference)	0.90 (0.75,1.08)	0.84 (0.64,1.10)	0.95 (0.72,1.24)	0.93 (0.64,1.35)	0.97 (0.69,1.38)	>0.99
	Model 2^2^	1.0 (reference)	0.92 (0.76,1.10)	0.85 (0.65,1.11)	0.96 (0.73,1.27)	0.93 (0.64,1.35)	0.97 (0.69,1.38)	0.99
	Model 3^3^	1.0 (reference)	0.92 (0.77,1.11)	0.85 (0.64,1.11)	0.96 (0.73,1.26)	0.91 (0.63,1.32)	0.97 (0.68,1.38)	0.92
**Colon cancer**	No of Cases (n = 529)	208	166	50	47	27	31	
	Model 1^1^	1.0 (reference)	0.85 (0.69,1.05)	0.76 (0.56,1.05)	0.81 (0.59,1.12)	0.94 (0.63,1.42)	1.02 (0.69,1.49)	0.82
	Model 2^2^	1.0 (reference)	0.86 (0.70,1.06)	0.77 (0.56,1.05)	0.81 (0.59,1.12)	0.94 (0.62,1.42)	1.00 (0.68,1.47)	0.89
	Model 3^3^	1.0 (reference)	0.87 (0.70,1.07)	0.77 (0.56,1.06)	0.81 (0.58,1.12)	0.92 (0.61,1.39)	1.00 (0.68,1.47)	0.94
**Rectal cancer**	No of Cases (n = 155)	49	54	18	22	6	6	
	Model 1^1^	1.0 (reference)	1.12 (0.75,1.65)	1.14 (0.66,1.97)	1.52 (0.91,2.54)	0.90 (0.38,2.14)	0.80 (0.34,1.88)	0.69
	Model 2^2^	1.0 (reference)	1.15 (0.77,1.70)	1.18 (0.68,2.05)	1.61 (0.96,2.70)	0.87 (0.36,2.08)	0.86 (0.36,2.02)	0.79
	Model 3^3^	1.0 (reference)	1.14 (0.77,1.71)	1.18 (0.68,2.06)	1.60 (0.95,2.71)	0.85 (0.35,2.05)	0.87 (0.36,2.05)	0.79
**Proximal colon cancer**	No of Cases (n = 243)	89	84	21	21	12	16	
	Model 1^1^	1.0 (reference)	1.04 (0.77,1.41)	0.78 (0.48,1.26)	0.87 (0.54,1.41)	1.00 (0.54,1.84)	1.24 (0.72,2.14)	0.55
	Model 2^2^	1.0 (reference)	1.04 (0.77,1.41)	0.79 (0.49,1.28)	0.89 (0.55,1.44)	1.02 (0.55,1.88)	1.20 (0.70,2.07)	0.62
	Model 3^3^	1.0 (reference)	1.05 (0.78,1.43)	0.81 (0.50,1.31)	0.88 (0.54,1.44)	1.00 (0.54,1.86)	1.19 (0.68,2.05)	0.68
**Distal Colon cancer**	No of Cases (n = 189)	72	57	23	16	10	11	
	Model 1^1^	1.0 (reference)	0.83 (0.58,1.17)	0.99 (0.61,1.60)	0.74 (0.43,1.28)	1.08 (0.55,2.10)	0.99 (0.52,1.89)	0.87
	Model 2^2^	1.0 (reference)	0.84 (0.59,1.19)	1.00 (0.62,1.62)	0.74 (0.43,1.30)	1.09 (0.55,2.13)	0.99 (0.52,1.89)	0.88
	Model 3^3^	1.0 (reference)	0.84 (0.59,1.20)	0.99 (0.61,1.61)	0.73 (0.42,1.28)	1.07 (0.54,2.11)	0.97 (0.51,1.86)	0.94

1 adjusted for age (in months).

2 adjusted for age (in months), smoking before age 30 (0, 1–4, 5–10, or >10 pack-years), history of colorectal cancer in a parent or sibling (yes, no), history of colonoscopy or sigmoidoscopy (yes, no), regular aspirin use (yes, no), body mass index (<25, 25–<30, ≥30 kg/m^2^), physical activity (<3, 3–<27, ≥27 MET-hrs/wk).

3 adjusted for age (in months), factors listed in model 2, consumption of processed meat (quintiles), consumption of beef, pork, or lamb as a main dish (quintiles), alcohol consumption (0–<5, 5–<10, 10–<15, or ≥15 g/d), multivitamin use (yes, no), energy-adjusted total calcium intake (quintiles), total folate intake (quintiles), and total vitamin D intake (quintiles).

With regard to ferritin, we conducted regression analyses in which we regressed the medians of each of blood donation category (i.e., 0, 3, 7.5, 15, 25, 35) on serum ferritin levels. For mortality analysis, person-time was calculated from the date of the baseline questionnaire until the date of death or the end of follow-up (January 1, 2008), whichever occurred first. We used a Cox proportional hazards regression model [23] to calculate the RRs (95% CIs).

All statistical analyses were two-sided with a *P*-value less than 0.05 indicating significance. We conducted all analyses using the SAS software (SAS Institute, Inc., Version 9.2, Cary, NC).

## Results

A total of 684 incident colorectal cancer cases were documented during 504,122 person-years of follow-up. As shown in table 1, although differences in colorectal cancer risk factors between frequent blood donors and non-donors were quite modest, blood donation was associated with serum ferritin levels. Among the 305 men, mean ferritin levels across the categories of number of blood donations were: 178 µg/L for no donations (n = 90); 173 µg/L for 1 to 4 donations (n = 110); 162 µg/L for 5 to 9 donations (n = 36); 129 µg/L for 10 to 19 donations (n = 34); 102 µg/L for 20 to 29 donations (n = 12); and 98 µg/L for ≥30 blood donations (n = 23) (Table 1). The regression models showed that 1 unit change in blood donation (i.e., 1 donation) results in significant 2.6 µg/L lower levels of serum ferritin (*P* = 0.002). Results were essentially the same when we further adjusted for lifestyle and other dietary factors (data not shown).

We found no significant association between the number of blood donations and incidence of colorectal cancer overall or by any sub-site (Table 2). The age-adjusted relative risk (RR) was 0.97 (95%CI: 0.68, 1.38, *P* for trend = 0.92) for the highest blood donation group (i.e., ≥30) compared with the non-donors. The RRs were essentially unchanged after adjustment for non-dietary or dietary factors (Table 2). Further adjustment for iron supplement use, total iron intake, and heme iron intake (each factor was added separately to the model) did not change the observed null associations. In addition, stratified analysis by total iron intake and heme iron intake (<median, ≥median) yielded similar null results (data not shown). Further, as shown in Table 1, only 2–3% men took iron supplements in 1992, which limited our ability to restrict our analysis among the iron-supplement user group (n = 28 colorectal cancer cases). The null associations were not modified by age (<65, ≥65 y), alcohol consumption (<5 g/d, ≥5 g/d), endoscopy screening (no, yes), body mass index (<25, ≥25 kg/m^2^), physical activity (<30, ≥30 MET-hrs/wk), and family history of colorectal cancer (no, yes) (all *P*-values for interaction ≥0.15; data not shown). We conducted sensitivity analyses to further adjustment for chronic disease such as cardiovascular disease, hypertension, and history of diabetes or restricted to men without gastrointestinal bleeding and inflammatory conditions such as myocardial infarction, stroke, coronary artery bypass graft angina or rheumatoid arthritis and results were essentially unchanged (data not shown). Given that approximately 70–80% of colorectal cancer in developed countries is colon cancer, [25] we classified 78 colorectal cancer cases with unknown information on sub-site into the colon cancer. Sensitivity analysis excluding these cases did not change the results (data not shown).

A total of 224 deaths from colorectal cancer (fatal colorectal cancer) were identified. The number of lifetime blood donations was not associated with colorectal cancer mortality. Similar to the results from analyses of colorectal cancer incidence, age-adjusted results were essentially the same as the multivariable-adjusted results (Table 3).

**Table 3:** The relative risks of fatal colorectal cancer according to lifetime number of blood donations in the Health Professionals Follow-up Study (1992–2008).

		Number of blood donations					P for trend
		0	1–5	6–9	10–19	≥20	
**Colorectal cancer**	No of deaths	85	76	21	18	24	
	Model 1^1^	1.0 (reference)	1.00 (0.73,1.36)	0.88 (0.54,1.44)	0.80 (0.50,1.34)	1.12 (0.71,1.78)	0.97
	Model 2^2^	1.0 (reference)	0.99 (0.72,1.35)	0.93 (0.57,1.51)	0.84 (0.50,1.41)	1.14 (0.71,1.81)	0.84
	Model 3^3^	1.0 (reference)	0.99 (0.72,1.36)	0.93 (0.57,1.51)	0.85 (0.50,1.42)	1.14 (0.72,1.83)	0.82

1 adjusted for age (in months).

2 adjusted for age (in months), smoking before age 30 (0, 1–4, 5–10, or >10 pack-years), history of colorectal cancer in a parent or sibling (yes, no), history of colonoscopy or sigmoidoscopy (yes, no), regular aspirin use (yes, no), body mass index (<25, 25–<30, ≥30 kg/m^2^), physical activity (<3, 3–<27, ≥27 MET-hrs/wk).

3 adjusted for age (in months), factors listed in model 2, consumption of processed meat (quintiles), consumption of beef, pork, or lamb as a main dish (quintiles), alcohol consumption (0–<5, 5–<10, 10–<15, or ≥15 g/d), multivitamin use (yes, no), energy-adjusted total calcium intake (quintiles), total folate intake (quintiles), and total vitamin D intake (quintiles).

## Discussion

Frequent blood donation, a marker of body iron stores, was not associated with colorectal cancer risk in this prospective study of 35,121 middle-aged to elderly U.S. male health professionals. Our findings do not support an important association between body iron stores, reflected by serum ferritin levels, and colorectal cancer incidence and mortality in men.

Limitations of our studies warrant consideration. We only queried the information on the number of blood donations once and when the donations were made is uncertain. Thus, the misclassification of the number of blood donations is possible and how the timing of blood donations may have influenced the current results is uncertain. In addition, our study population is men only and we lack data on blood donation in premenopausal women, who tend to have much lower ferritin levels due to menstrual bleeding and pregnancy. [24], [26], [27] Moreover, although our study is large overall, we had limited numbers for analysis of mortality as well as analysis of incidence among certain sub-sites. Further, the healthy donor effect might explain the lack of an association but regular blood donors in these male professionals were generally comparable with non-donors with respect to characteristics shown in Table 1.

Strengths of this study include its large size, prospective design, long follow-up time and high follow-up rate. Because of the observed approximate 2-fold difference in serum ferritin levels by frequency of blood donations, the iron-colorectal cancer hypothesis can be reasonably tested using blood donation as a surrogate. In addition, comparable prevalence of endoscopy screening practice across categories of blood donation group and the null results from mortality analysis appear to decrease concerns that the observed results were influenced by detection bias. Moreover, although it cannot be totally ruled out, unmeasured confounding seems unlikely to have totally cancelled or reversed our results because the age-adjusted and multivariable-adjusted results were essentially the same.

Iron is vital for human health and iron deficiency can cause anemia. [2], [9] But iron is a pro-oxidant and high iron levels can form reactive oxygen species and free radicals and cause DNA damage. [1], [2] In addition, tumor cells grow better in an iron-rich environment [28] suggesting a potential role of iron in neoplastic cell proliferation. Thus, excessive iron may increase cancer risk. The hypothesis of depletion of iron reducing colorectal cancer risk [29] has been examined in many epidemiologic studies of biochemical markers, dietary intakes, and genetic mutations, but the results have been conflicting. Human observational studies of colorectal cancer risk in relation to endogenous body iron stores, [3], [5], [9], [12] measured by serum iron, transferrin saturation and total iron binding capacity yielded mixed results. Some studies found suggestions of an inverse association, [3] but others reported no relation [5], [12] or even the possibility of an increased risk. [9] Although not totally understood, one single time measurement of iron status such as serum iron and transferrin saturation may not reliably correlate with the body iron stores, [2] which may partly explain the inconsistent results. In addition, results from studies of exogenous intakes of iron [4], [6], [7], [10], [12], [13], [30] including dietary iron and heme iron, the more bioavailable form of iron compared with other forms of iron, have also been inconclusive. A recent meta-analysis of five studies of heme iron reported a modest positive association between heme iron intake and colon cancer risk (highest vs. lowest quintile, RR = 1.18, 95%CI: 1.06, 1.32; *P* value for heterogeneity  = 0.18). [31] Heme iron, mainly found in animal foods, has been shown to have cytotoxic effects in rats [32] and to increase formation of endogenous intestinal N-nitroso compounds, [33] established human carcinogens. Given that iron absorption is generally tightly regulated, [2] suggestive positive associations with heme iron indicated that luminal exposure to the unabsorbed iron might play a role in colorectal carcinogenesis. If elevated body iron stores is a risk factor for colorectal cancer, individuals with hereditary hemochromatosis (HFE) characterized by iron overload would have a higher risk. However, positive associations with mutations in HFE, the gene for hereditary hemochromatosis, were reported in some studies, [34], [35] but not in others. [36], [37], [38] In addition, investigations of iron related biomarkers, intake, and gene mutations in relation to risk of adenomas, [12], [35], [36] precursor lesions of colorectal cancer also yielded mixed results. Collectively, the inconsistency appeared not to be totally explained by differences in study design, iron related variables examined, or adjustment for confounding factors and the effect of endogenous or extraneous iron on colorectal cancer risk remains uncertain. Of note, based on an average of 4.5 years of follow-up, risk of colorectal cancer was lower in an iron reduction group (4 vs. 9 cases in control group) in a recent clinical trial of phlebotomy for reduction of iron stores among older most male patients with advanced peripheral arterial disease. [39] The trial was not originally designed to compare cancer risk and the lower range of the iron levels among those patients who did not develop cancer cannot be generalized to general population to reduce cancer incidence.

Given that blood donation is good marker of serum ferritin as shown here and in previous studies, [14], [22] the iron-colorectal cancer hypothesis can also be tested by examining risk of colorectal cancer between frequent blood donors versus non-regular donors. Low risk of cancer incidence and mortality has been reported among blood donors [16], [17], [18] but the results may reflect the healthy lifestyle and good health condition of the donor groups. [19] Recently, one study used a nested case-control study design and investigated the possible effects of iron loss that resulted from blood donation in a cohort of Swedish and Danish blood donors. [19] They observed a suggestive trend (*P* trend <0.001) of decreasing risk for cancers of liver, lung, colon, stomach, and esophagus combined among men with a latency of 3–7 years comparing the lowest to the highest estimated iron loss (OR = 0.70, 95%CI:0.58,0.84). [19] However, as recognized by the authors, how the observed results were affected by unmeasured confounding factors such as smoking, alcohol consumption, body mass index, physical activity, and occupation exposures remains unclear. [19] They concluded that repeated blood donation was not associated with increased or decreased risk of cancer overall. [19] In addition, no separate estimates were reported for colon cancer in that study due to small numbers, which does not allow direct comparison with our results. Nonetheless, the null associations observed in our study are generally consistent with their conclusion. In addition, in the same cohort examined here, intakes of iron and heme iron were not significantly associated with colorectal cancer risk. [13].

In summary, frequent blood donation was not significantly associated with colorectal cancer incidence and mortality among U.S. men in this study. The results indirectly suggested that body iron stores may not play an important role in colorectal carcinogenesis.
